# Immunogenicity and Efficacy of a Feed-Based Bivalent Vaccine against Streptococcosis and Motile Aeromonad Septicemia in Red Hybrid Tilapia (*Oreochromis* sp.)

**DOI:** 10.3390/ani13081346

**Published:** 2023-04-14

**Authors:** Nur Shidaa Mohd Ali, Mohd Zamri Saad, Mohammad Noor Amal Azmai, Annas Salleh, Zarirah Mohamed Zulperi, Tilusha Manchanayake, Muhammad Amir Danial Zahaludin, Lukman Basri, Aslah Mohamad, Ina Salwany Md Yasin

**Affiliations:** 1 Laboratory of Aquatic Animal Health and Therapeutics, Institute of Bioscience, Universiti Putra Malaysia, Serdang 43400, Selangor, Malaysia; nurshidaamohdali@gmail.com (N.S.M.A.);; 2 Department of Biology, Faculty of Science, Universiti Putra Malaysia, Serdang 43400, Selangor, Malaysia; 3 Department of Veterinary, Laboratory Diagnosis, Faculty of Veterinary Medicine, Universiti Putra Malaysia, Serdang 43400, Selangor, Malaysia; 4 Department of Aquaculture, Faculty of Agriculture, Universiti Putra Malaysia, Serdang 43400, Selangor, Malaysia

**Keywords:** red hybrid tilapia, streptococcosis, motile Aeromonad septicemia, feed-based bivalent vaccine, immuno-protective

## Abstract

**Simple Summary:**

Vaccines play a pivotal role in the control of infectious diseases. This study investigated the immuno-protective efficacy of a newly developed feed-based bivalent vaccine against streptococcosis and motile Aeromonad septicemia (MAS) in red hybrid tilapia. The feed-based bivalent vaccine was found enhanced mucosal and systemic immunities in red hybrid tilapia. The feed-based bivalent vaccine also improved relative percentage survival (RPS) in red hybrid tilapia through protections against infections by *Streptococcus agalactiae* and *Aeromonas hydrophila* and partial cross-protections against *Streptococcus iniae* and *Aeromonas veronii*.

**Abstract:**

Streptococcosis and motile Aeromonad septicemia (MAS) are the main bacterial diseases in tilapia culture worldwide, causing significant economic losses. Vaccination is an effective method of preventing diseases and contributes to economic sustainability. This study investigated the immuno-protective efficacy of a newly developed feed-based bivalent vaccine against streptococcosis and MAS in red hybrid tilapia. The feed-based bivalent vaccine pellet was developed by incorporating the formalin-killed *S. agalactiae* and *A. hydrophila* antigens into a commercial feed pellet with palm oil as the adjuvant. The bivalent vaccine was subjected to feed quality analyses. For immunological analyses, 900 fish (12.94 ± 0.46 g) were divided into two treatment groups in triplicate. Fish in Group 1 were unvaccinated (control), while those in Group 2 were vaccinated with the bivalent vaccine. The bivalent vaccine was delivered orally at 5% of the fish’s body weight for three consecutive days on week 0, followed by boosters on weeks 2 and 6. Lysozyme and enzyme-linked immunosorbent assays (ELISAs) on serum, gut lavage, and skin mucus were performed every week for 16 weeks. Lysozyme activity in vaccinated fish was significantly (*p* ≤ 0.05) higher than in unvaccinated fish following vaccination. Similarly, the IgM antibody levels of vaccinated fish were significantly (*p* ≤ 0.05) higher after vaccination. The bivalent vaccine provided high protective efficacy against *S. agalactiae* (80.00 ± 10.00%) and *A. hydrophila* (90.00 ± 10.00%) and partial cross-protective efficacy against *S. iniae* (63.33 ± 5.77%) and *A. veronii* (60.00 ± 10.00%). During the challenge test, fewer clinical and gross lesions were observed in vaccinated fish compared with unvaccinated fish. Histopathological assessment showed less severe pathological changes in selected organs than the unvaccinated fish. This study showed that vaccination with a feed-based bivalent vaccine improves immunological responses in red hybrid tilapia, and thus protects against streptococcosis and MAS.

## 1. Introduction

Tilapia (*Oreochromis* spp.) is key freshwater fish that is intensively cultured worldwide. Global production for tilapia is forecasted to grow by 3.7% in 2022, breaking the six million-metric-ton barrier [1]. Tilapia, including the Mozambique tilapia (*Oreochromis mossambicus*), the Nile tilapia (*O. niloticus*), the blue tilapia (*O. aureus*), and different hybrids of the red tilapia (*Oreochromis* sp.), is the most commonly cultured fish worldwide [2]. In Malaysia, the red hybrid tilapia has become the dominant aquaculture species (>90%) due to high consumer demand [3,4]. Although tilapia production is expanding worldwide, it also faces enormous bacterial and virus diseases such as streptococcosis, motile Aeromonad septicemia (MAS), and Tilapia Lake virus (TiLV) that have led to severe economic losses [5].

Among the bacterial diseases, streptococcosis and MAS are primary barriers for sustainable tilapia production [6]. *Streptococcus agalactiae* and *S. iniae* have been recognized as the main culprits of streptococcosis [7,8], while *Aeromonas hydrophila* and *A. veronii* are the main agents for MAS disease [9,10]. Nevertheless, the mortality of cultured tilapia is not only caused by a single infection; it can be caused by the co-infection of different microorganisms. Several cases of co-infections include *Streptococcus* and *Aeromonas* [11], *Streptococcus* and *Francisella* [12], and *Aeromonas* and *Flavobacterium* [13].

The most common approach in controlling bacterial-based tilapia diseases is the use of antibiotics [14]. However, the use of antibiotics is not being encouraged due to the development of antibiotic-resistant strains [15,16]. Moreover, severe complications arise through the development of antibiotic-resistant bacteria and accumulations of antibiotic residue in the food chain [17] that could harm humans and the environment [18]. Thus, vaccination has been suggested as an alternative to preventing outbreaks of bacterial infection in aquaculture [19,20].

Fish vaccines can be administered through immersion, injection, and oral methods [21]. Oral vaccination is highly preferred nowadays since it is easy and relevant to all sizes of fish, less stressful to fish, decreases the labor cost, and allows vaccination booster during the culture period [22]. Recently, a novel feed-based bivalent vaccine has been developed to control *Streptococcus* and *Aeromonas* infections [23]. This approach has offered practical and easy application while reducing workload compared with other vaccination methods. In 2020, a feed-based bivalent vaccine containing formalin-killed *S. iniae* and *A. hydrophila* with the addition of palm oil was developed [23,24]. The present study investigated the immuno-protective efficacy of a newly developed feed-based bivalent vaccine against streptococcosis and MAS in red hybrid tilapia.

## 2. Materials and Methods

### 2.1. Red Hybrid Tilapia (Oreochromis sp.)

Red hybrid tilapias (*n* = 900) with an average body weight of 10.30 ± 0.35 g and length of 9.15 ± 0.10 cm were obtained from a local fish farm (Razham AGRO Trading, Beranang, Selangor, Malaysia) and were transferred to the Fish Hatchery Unit, Laboratory of Aquatic Animal Health and Therapeutics (AquaHealth), Institute of Bioscience (IBS), Universiti Putra Malaysia, Serdang, Selangor, Malaysia. The fish were acclimatized under laboratory conditions for 30 days before 10 fish were randomly selected and screened for bacterial diseases. The fish blood, kidney, liver, and spleen were subjected to bacterial isolation. The fish were found to be healthy, with no bacteria found in any sample. The water quality was maintained as follows: temperature (27–28 °C), dissolved oxygen (5.82–6.67 mg/L), pH (7.21–7.74), and ammonia-nitrogen (<0.01 mg/L).

### 2.2. Bacterial Pathogens and Culture Conditions

The bacterial pathogens, namely, *S. agalactiae* [25], *A. hydrophila* [26], *S. iniae* [27], and *A. veronii* [28], were initially isolated from infected red hybrid tilapia and were stored at AquaHealth, UPM. Virulence of the bacterial pathogens was revived according to Koch’s postulate protocol. Briefly, 200 µL of bacterial culture broth grown in tryptone soya broth (TSB; Oxoid, Hampshire, England) was administered by intraperitoneal (IP) injection into a healthy red hybrid tilapia based on the respective median lethal dose (LD_50_). The pathogens were recovered from the infected fish organs, including the brain, kidney, liver, and spleen, at 24 h post-infection. The obtained bacteria were streaked on blood agar plate and subjected to colony polymerase chain reaction (PCR) for identification [24]. For routine use, bacterial pathogens were cultured on tryptone soya agar (TSA; Oxoid, Hampshire, England) and incubated at 30 °C for 18 h.

### 2.3. Feed-Based Bivalent Vaccine Preparation

The feed-based bivalent vaccine was prepared based on Monir et al. [23]. Briefly, *S. agalactiae* and *A. hydrophila* were cultured separately on TSA and incubated at 30 °C for 18 h. The cultures were transferred from TSA into TSB and incubated at 30 °C for 18 h with shaking at 150 rpm. After incubation, the bacterial concentration was determined [29] and adjusted to 6.4 × 10^9^ CFU/mL. The bacterial cells were then killed by adding 0.5% (*v*/*v*) of formalin and kept at 4 °C overnight [30]. To ensure that all respective bacteria were killed, the bacterial suspension was streaked on TSA and incubated at 30 °C for 18 h. The formalin-killed *S. agalactiae* and *A. hydrophila* were collected by centrifugation (6000× *g*, 8 min, 4 °C). To wash out the remaining medium and formalin from the broth cultures, bacterial pellets were washed four times using sterile 1× phosphate-buffered saline (PBS) buffer at pH 7 followed by centrifugation (6000× *g*, 15 min, 4 °C). The bacterial pellets of formalin-killed *S. agalactiae* and *A. hydrophila* were re-suspended in 100 mL of sterile 1× PBS buffer. The individual bacterial suspension was emulsified with 10% palm oil before sterile 1× PBS buffer was added into the mixture until 1 L.

The mixture was then added into 1 kg of a commercial tilapia feed (Star Feed, Klang, Selangor, Malaysia) powder and was mixed thoroughly using a mixer machine (Golden Bull-B10-A Universal Mixers, Johor Bahru, Malaysia) before being loaded into a mini pelleting machine (Golden Avill, Guangdong Province, China) to form pellets of 4 mm × 2 mm in size. The pellets were dried in a hot air oven (Memmert, Schwabach, Germany) for 12 h at 28 °C. To prepare control feed, only PBS and 10% palm oil were used. All feed pellets were stored at room temperature (25 ± 2 °C) until further use.

### 2.4. Feed Quality Analyses

#### 2.4.1. Proximate Analysis

The feed used in this study was subjected to proximate analysis, which was conducted by UNIPEQ Sdn. Bhd., Universiti Kebangsaan Malaysia, Malaysia. The carbohydrate was detected using in-house method No: STP/Chem/A06 according to the Pomeranz *Food Analysis: Theory and Practice*, 2nd ed., page 637. The energy was calculated using in-house method No: STP/Chem/A01 according to Pearson’s *The Chemical Analysis of Foods*, 6th ed., page 578. Moisture was analyzed using in-house method No: STP/Chem/A04 according to the AOAC, 20th ed.: 950.46. Total protein was measured using in-house method No: STP/Chem/A03 according to the AOAC, 20th ed.: 981.10. Total ash was analyzed using in-house method No: STP/Chem/A05 according to the AOAC, 20th ed.: 923.03, and total fat was measured using in-house method No: STP/Chem/A02 according to the AOAC, 20th ed.: 991.36.

#### 2.4.2. Palatability Test

Feed acceptability by fish was determined based on the established method of Dong et al. [31]. The red hybrid tilapia (*n* = 90) were split into three groups in triplicate. The glass tank (30 L) was filled with water and randomly distributed with 10 fish (average body weight 15.10 ± 0.72 g) in each glass tank. Before feeding, fish were starved for 24 h. Group 1 was fed commercial tilapia pellets, Group 2 was fed control pellets, and Group 3 was fed vaccine pellets. Fish from each tank were fed with 2 g of pellets. The uneaten pellets were collected one hour later. The palatability based on the ingestion ratio (Ri) was calculated using the formula:

$$\mathrm{Ri}=\frac{Weight\;of\;pellet\;ingested\;\left(g\right)}{Weight\;of\;pellets\;fed\;\left(g\right)}$$

#### 2.4.3. Physical Stability in Water

Stability of the pellets (commercial, control, and vaccine pellets) in water was determined by the static water procedure [32]. A 250 mL conical flask with 100 mL of water was used to leach 2 g of each pellet in triplicate for the required immersion periods of 0, 1, 2, 3, 4, 5, 6, and 7 h. The immersed pellet was further filtered using Whatman filter paper (No 1) once the necessary leaching period had passed. The recovered and original pellet samples were dried in the oven at 100 °C for 24 h and were analyzed for pellet stability based on the dry matter retention using the following formula:

$$Dry\;matter\;retention=\left(\frac{Feed\;remaining\;\left(g\right)}{Initial\;feed\;\left(g\right)}\right)\times 100$$

#### 2.4.4. Vaccine Safety Test

To test the safety of the vaccine, 30 fish were fed with the bivalent vaccine containing formalin-killed *S. agalactiae* and *A. hydrophila* whole-cell antigens at a concentration of 10^9^ cfu/fish. Then, they were observed for 14 days for adverse side effects, which included behavior changes, such as lethargy, loss of appetite, aggression, and isolation; color changes; mortality; or other clinical signs related to vaccination [33]. A safe vaccine would not produce these changes.

#### 2.4.5. Growth Performance

Red hybrid tilapia (*n* = 102) with an average body weight of 10.30 ± 0.35 g and length of 9.15 ± 0.10 cm were divided into two treatment groups. Group 1 was the unvaccinated (control) and Group 2 was vaccinated. The fish were cultured individually in 30 L glass tanks in triplicates. Fish were fed twice daily for 16 weeks with a commercial tilapia pellet. However, in weeks 0, 2, and 6, fish of Group 1 were fed with the control pellet, while Group 2 was fed the vaccine pellet for three days. The weight and length of fish were measured weekly during the 16-week study period. The specific growth rate (SGR) and feed conversion ratio (FCR) were calculated using the following equations:

$$Total\;weight\;gain\;\left(wg\right)=Final\;body\;weight\;\left(g\right)-Initial\;body\;weight\;\left(g\right)$$

$$SGR=100\times \left(\frac{\left[Ln\left(Final\;body\;weight\right)-Ln\;\left(Initial\;body\;weight\right)\right]}{Duration}\right)$$

$$FCR=\frac{Feed\;intake\;\left(g\right)}{Weight\;gain\;\left(g\right)}$$

### 2.5. Vaccination Trial

#### Experimental Design

The study was performed at the Hatchery Unit, AquaHealth, IBS, UPM, Malaysia, in accordance with the Malaysian Code of Practice for The Care and Use of Animals for Scientific Purposes. The Institutional Animal Care and Use Committee, UPM, approved the study (approval number #UPM/IACUC/AUP-R076/2019).

After acclimatization, the red hybrid tilapia (*n* = 900), with an average body weight of 12.94 ± 0.46 g and length of 13.10 ± 0.72 cm, were transferred randomly into six fiberglass tanks, and divided into two experimental groups in triplicates. Each tank capacity was 500 L, containing 150 fish per tank with a stocking density of 53 fish/m^3^. On the vaccination weeks (weeks 0, 2, and 6), Group 1 (control; *n* = 450 fish) was fed a control pellet, while Group 2 (vaccinated; *n* = 450 fish) was fed the vaccine pellet. Before vaccination, fish were starved for 24 h. Both pellets were delivered orally on day 0 at 5% body weight, three times daily for three consecutive days. Booster doses were similarly administered in weeks 2 and 6. For other, non-vaccination weeks, fish were fed normal commercial pellets throughout the 16-week experimental period.

**Figure 1:**
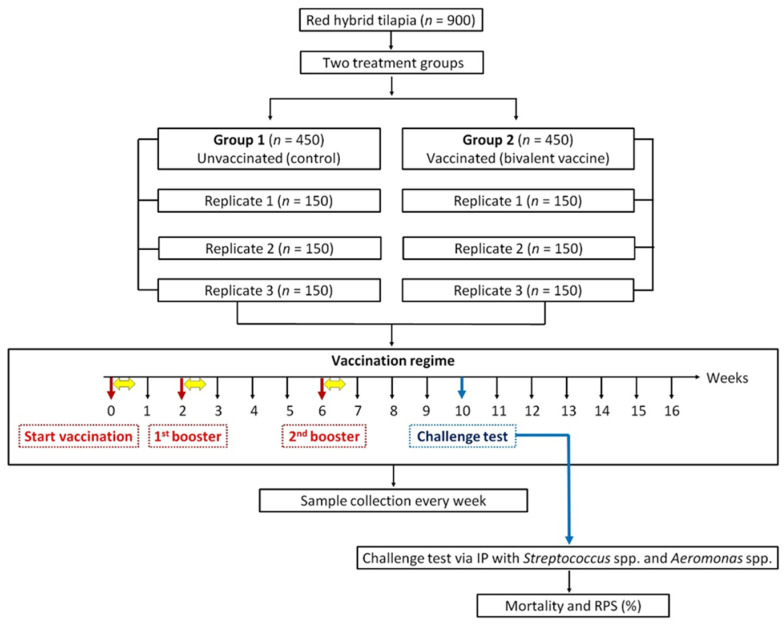
The experimental design flow chart used in this study. Fish in Group 1 were fed with the control pellets (PBS buffer + 10% palm oil) and acted as an unvaccinated (control) group. Fish in Group 2 were fed with the feed-based bivalent vaccine (whole-cell bacterial antigens + PBS buffer + 10% palm oil) and acted as a vaccinated group. Vaccination was delivered orally at 5% of the fish’s body weight for three consecutive days (yellow arrow) on weeks 0, 2, and 6. Samples (serum, gut lavage, and skin mucus) were collected every week for 16 weeks to assess the fish’s immunological response. Challenges via intraperitoneal injection (IP) with *S. agalactiae*, *S. iniae*, *A. hydrophila*, and *A. veronii* were conducted at week 10. The mortality and relative percentage survival (RPS) were observed for 14 days after the challenge test.

On week 10, fish were challenged intraperitoneally with *S. agalactiae*, *S. iniae*, *A. hydrophila*, and *A. veronii*. The mortality and RPS (%) were observed for 14 days. The procedure of the experiment is summarized in Figure 1. Throughout the experimental period, water quality was maintained at a temperature of 30.95 ± 2.72 °C, dissolved oxygen of 6.12 ± 1.39 mg/L, pH of 7.35 ± 1.42, and ammonia–nitrogen of 0.01 ± 0.001 mg/L.

### 2.6. Immunological Analyses

#### 2.6.1. Sample Collection

At weekly intervals, samples of serum, gut lavage, and skin mucus were taken from 6 fish/groups. Prior to sample collection, the selected fish were anesthetized with MS-222 at the dose of 10^5^ mg/L [24]. Approximately 0.8 mL of blood was collected from each fish through the caudal vein and transferred into a 1.5 mL vacutainer plain tube. All blood samples were kept at 4 °C overnight to allow blood clotting. The serum was extracted by centrifugation at 3000× *g* for 3 min at 4 °C and stored at −20 °C until further used.

For gut lavage collection, approximately 10 cm of the gut was collected from each fish. The collected gut was infused with 1 mL of sterile 1 × PBS containing 0.02% (*w*/*v*) of sodium azide (Sigma Aldrich, St. Louis, MO, USA) and gently massaged with fingers before the fluid was collected into 1.5 mL microcentrifuge tube. The gut lavage fluid was kept at 4 °C overnight and further subjected to centrifugation at 3000× *g* for 3 min at 4 °C. The gut lavage samples were stored at −20 °C until further used.

Skin mucus was sampled using a sterile cotton stick. The cotton stick was swabbed 10 times on one side of the fish’s body from the head to the caudal fins [34]. Next, the swab cotton stick was dipped into 1 mL sterile 1 × PBS containing 0.02% (*w*/*v*) of sodium azide before the skin mucus samples were kept overnight at 4 °C, and subjected to centrifugation at 3000× *g* for 3 min at 4 °C. The skin mucus samples were stored at −20 °C until further use.

#### 2.6.2. Lysozyme Assay

The lysozyme activity was determined using a turbidimetric assay [35]. In total, 25 µL of samples (serum, gut lavage, and skin mucus) was placed separately into the wells of a microplate. Then, 75 µL of substrate containing 0.2 mg of *Micrococcus lysodeikticus* lyophilized cell (Sigma-Aldrich, St. Louis, MO, USA)/mL PBS at pH 6.3 was added. The absorbance was measured at 450 nm after 1 min and 60 min incubation with gentle shaking at room temperature (25 ± 2 °C) using a Multiskan spectrum microplate reader (Thermo Fisher Scientific, Waltham, MA, USA). A unit of lysozyme activity was defined as the amount of enzyme causing a decrease in absorbance of 0.001 per min, and expressed as a U/mg unit.

#### 2.6.3. Enzyme-Linked Immunosorbent Assay (ELISA)

The IgM antibody titers against *S. agalactiae*, *A. hydrophila*, *S. iniae*, and *A. veronii* in the serum, gut lavage, and skin mucus of red hybrid tilapia were determined using ELISAs [23,36]. Coating antigens were prepared by individually culturing all pathogens in TSB and incubated overnight at 30 °C with shaking at 150 rpm. The bacterial concentration was adjusted to 10^5^ CFU/mL using the standard plate count method. Then, the coating antigen (100 μL) was introduced into the microtiter plates and kept at 4 °C for 24 h before being washed twice with PBST (PBS + 0.05% Tween 20). Next, 200 µL of 1% bovine serum albumin (BSA) diluted in PBS was introduced into the plate, and incubated at 37 °C for 1 h. Subsequently, 100 µL of serum, gut lavage, and mucus in PBS was transferred into the reaction well and incubated at 37 °C for 1 h. The unbounded antibodies were removed by washing three times with PBST. Then, 100 μL of goat anti-tilapia hyperimmune serum (Aquatic Diagnostics Ltd., Oban, Scotland, UK) diluted at 1:10,000 was added and further incubated at 37 °C for 1 h before 100 μL of conjugated rabbit anti-goat IgM horseradish peroxidase (Nordic MUbio, Susteren, The Netherland) diluted at 1:10,000 was added and incubated at 37 °C for 1 h. After being washed with PBST three times, 100 µL of TMB substrate solution (Thermo Fisher Scientific, Waltham, MA, USA) was added into the reaction wells to detect the bound conjugate. The reaction was stopped by adding 0.2 mol/L sulfuric acids (Sigma Aldrich, St. Louis, MO, USA). The absorbance was measured at 450 nm using a Multiskan spectrum microplate reader.

#### 2.6.4. Challenge Trial

At week 10 post-vaccination, all fish were challenged intraperitoneally with live *S. agalactiae* at 1.8 × 10^3^ cfu/mL [37], *S. iniae* at 1.5 × 10^3^ cfu/mL [27], *A. hydrophila* at 2.0 × 10^4^ cfu/mL [38,39], and *A. veronii* at 1.8 × 10^4^ cfu/mL [40]. Intraperitoneal injection-based infection represents an efficient route of infection that shortens the time to develop signs of disease. The challenge was conducted in triplicate in 30 L glass tanks containing 10 fish in each tank. Prior to the challenge, the fish were anesthetized with 10^5^ mg/L of MS-222 (Sigma Aldrich, St. Louis, MO, USA). Both control and vaccinated groups were challenged with the respective live pathogen based on the LD_50_. Following challenge, the fish were monitored daily for clinical signs and abnormalities. The mortality and RPS were calculated using the following formula:

$$Cumulative\;percent\;mortality\;\left(CPM\right)=\frac{Number\;of\;fish\;mortality}{Total\;number\;of\;fish}\times 100$$

$$RPS=1-\left(\frac{Average\;CPM\;in\;the\;vaccinated\;group}{Average\;CPM\;in\;the\;control\;group}\times 100\right)$$

Dead fish were dissected immediately, and swabs from the brain, eye, spleen, liver, kidney, and skin lesions were streaked onto blood agar and incubated at 30 °C for 18 h. The obtained isolates were identified using PCR [24]. The primer set 27F/1492R (27F: 5′-GAGTTTGATCCTGGCTCAG-3′ and 1492R: 5′-GGTTACCTTGTTACGACTT-3′) was used for 16S rRNA gene amplification with a length of approximately 1500 bp. The amplified PCR products were subjected to sequencing (Apical Scientific Sdn. Bhd., Selangor, Malaysia). The obtained sequences were analyzed and compared against the sequences in the National Centre for Biotechnology Information (NCBI) database using the nucleotide BLAST (blastn) program http://blast.ncbi.nlm.nih.gov (accessed on 20 June 2022).

### 2.7. Histopathological Assessment

Microscopic examinations were conducted to assess pathological changes at the histological level [41]. The brain, kidney, liver, and spleen samples of freshly dead fish were fixed in 10% buffered formalin solution, sectioned at 4 µm and stained with hematoxylin and eosin (H & E). The slides were observed under a light microscope (Moticam Pro 285A; Motic Incorporation Ltd., Kowloon, Hong Kong), photographed using a Moticam camera (Motic Incorporation Ltd., Kowloon, Hong Kong) and analyzed using MIP software (Microscopic Image Processing; Motic Incorporation Ltd., Kowloon, Hong Kong).

### 2.8. Statistical Analysis

Microsoft Excel (Microsoft, Redmond, WA, USA) was used to conduct the statistical data analyses, which included the *t*-test and one-way analysis of variance (ANOVA) with a Tukey–Kramer post hoc test for comparing control and treatment groups. A value of *p* ≤ 0.05 was regarded as statistically significant.

## 3. Results

### 3.1. Feed Quality Analyses

#### 3.1.1. Proximate Analysis

The proximate compositions of control and vaccine pellets are presented in Table 1. Notably, significant higher energy and total fat contents (*p* ≤ 0.05) were found in the bivalent vaccine pellet than in the commercial tilapia pellet. However, the proximate compositions of commercial tilapia and formulated vaccine pellets were within the range of the standard dietary for tilapia as provided by the FAO (2017).

#### 3.1.2. Palatability Test

**Figure 2:**
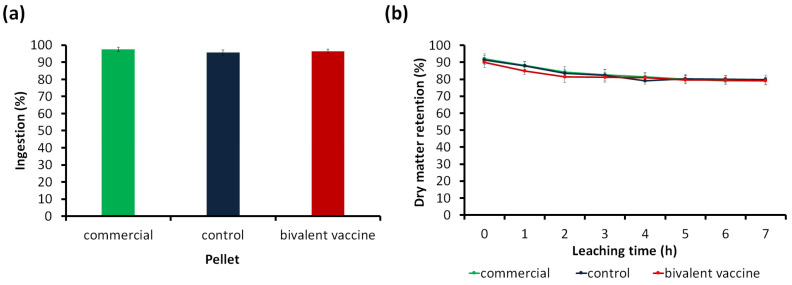
Palatability and physical stability of the pellets. (**a**) The ingestion percentage of commercial tilapia and formulated (control and bivalent vaccine) pellets; (**b**) the percentage of dry matter retention over leaching time for commercial tilapia and formulated (control and bivalent vaccine) pellets using the static water method. Results are the average triplicate ± standard deviation data (error bar). Insignificant different (*p* ≥ 0.05) between treatments.

The palatability of the commercial tilapia and formulated (control and bivalent vaccine) pellets are summarized in Figure 2a. 

The ingestion values (%) for commercial, control, and bivalent vaccine pellets were 97.49 ± 1.30%, 95.61 ± 1.54%, and 96.39 ± 1.27%, respectively, which were insignificant (*p* > 0.05) differences. This suggested that the formulated pellets did not affect the palatability of red hybrid tilapia.

#### 3.1.3. Physical Stability in Static Water Test

The physical stability in static water for the commercial tilapia and formulated pellets are shown in Figure 2b. The results revealed insignificant differences (*p* ≥ 0.05) between commercial tilapia, control, and bivalent vaccine pellets within 0–7 h of leaching. At the end of 7 h, all pellets appeared stable with 79.17% (commercial tilapia), 79.83% (control), and 79.00% (bivalent vaccine) dry matter.

#### 3.1.4. Growth Performance

**Figure 3:**
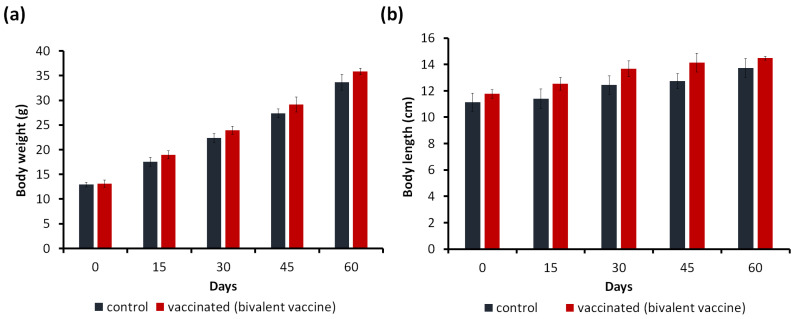
Effects of formulated pallets on the red hybrid tilapia’s growth performance. (**a**) Body weight and (**b**) body length of red hybrid tilapia after 60 days. Results are the average data in triplicate ± standard deviation (error bar). Insignificant differences (*p* ≥ 0.05) between control and vaccinated group.The maximum mean final weight was observed in the vaccinated (35.83 ± 1.20 g) compared with the unvaccinated (33.67 ± 0.98 g) groups (*p* ≥ 0.05).

The growth performance of the red hybrid tilapia following 60 days of feeding is summarized in Figure 3a,b.

Similarly, the maximum mean body length was observed in the vaccinated (13.7 ± 0.71 cm) and unvaccinated (14.5 ± 0.14 cm) groups (*p* ≥ 0.05). The weight gain values of the vaccinated and unvaccinated groups were 22.73 ± 1.83 g and 20.73 ± 0.97 g, respectively. The SGR was recorded higher in the vaccinated group, which was 1.68 ± 0.35% compared with the unvaccinated group (1.59 ± 0.49%). However, the results suggested that there were no significant differences (*p* ≥ 0.05) between the two treatment groups. The FCR values of the vaccinated and unvaccinated groups were 1.82 ± 0.10 g and 1.97 ± 0.07 g, respectively (Table 2).

### 3.2. Intraperitoneal Challenge Test

#### 3.2.1. Clinical Signs, Gross Lesions, and Bacteriology

The clinical sign and gross lesions during post-challenge with virulent *Streptococcus* spp. and *Aeromonas* spp. were observed as early as 12 h until 14 days. In most unvaccinated fish, death occurred as early as 12 h post-challenge. In contrast, death in the vaccinated fish started after 48 h. The clinical signs, particularly among unvaccinated fish, included erratic swimming, sluggish behavior, and in-appetence following challenge with *Streptococcus* spp. and *Aeromonas* spp. Meanwhile, the vaccinated fish only showed erratic swimming following challenge with *Streptococcus* spp. and *Aeromonas* spp.

**Figure 4:**
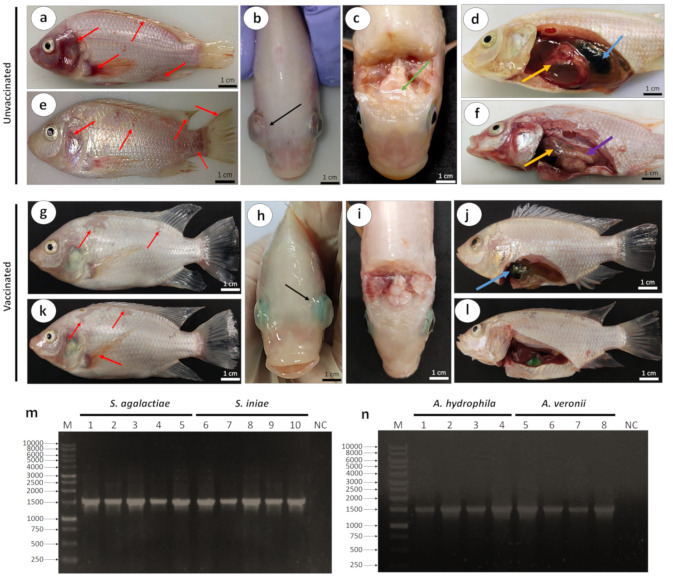
Gross lesions shown by red hybrid tilapia following challenge with *Streptococcus* spp. or *Aeromonas* spp. (**a**–**d**) The gross lesions shown by unvaccinated fish that were challenged with *Streptococcus* spp. (**e**,**f**) Gross lesions shown by unvaccinated fish following challenge with *Aeromonas* spp. (**a**) Hemorrhages in the skin of operculum, and the dorsal, caudal and pectoral fins (red arrow). (**b**) Bilateral exophthalmia (black arrow). (**c**) Softening of the brain (green arrow). (**d**) An enlarged liver (yellow arrow) and swelling of the gall bladder (blue arrow). (**e**) Hemorrhages in the skin of operculum, and the dorsal, caudal, and pectoral fins (red arrow). (**f**) Pale liver (purple arrow), an enlarged liver (yellow arrow). Picture (**g**–**l**) are gross lesions observed in vaccinated fish. (**g**) Hemorrhages affecting the scales (red arrow). (**h**) Bilateral exophthalmia (black arrow). (**i**) Normal brain. (**j**) Swelling of gall bladder (blue arrow). (**k**) Hemorrhages in the skin (scales and pectoral fin) (red arrow). (**l**) Normal liver and gall bladder. (**m**) Amplification of 16S rRNA gene sequence of *Streptococcus* spp. isolated from the eye (lanes 1 and 6), brain (lanes 2 and 7), spleen (lanes 3 and 8), liver (lanes 4 and 9), and kidney (lanes 5 and 10) of the infected dead fish. (**n**) Amplification of the 16S rRNA gene sequence of *Aeromonas* spp. isolated from a skin lesion (lanes 1 and 5), liver (lanes 2 and 6), kidney (lanes 3 and 7), and spleen (lanes 4 and 8) of the infected dead fish. Lane NC, negative control; lane M = 1 kb DNA ladder marker (Fermentas, Waltham, MA, USA).

Unvaccinated fish that were challenged with *Streptococcus* spp. showed external lesions such as severe hemorrhages in the skin of operculum, dorsal fin, caudal fin and pectoral fin (Figure 4a), and bilateral exophthalmia (Figure 4b). The internal organs showed softening of the brain (Figure 4c) and swollen livers and gall bladders (Figure 4d). Meanwhile, unvaccinated fish that were challenged with *Aeromonas* spp. showed the same external lesions, including hemorrhages in the skin of operculum and dorsal, caudal, and pectoral fins (Figure 4e). Pale and enlarged livers (Figure 4f) could be observed in the unvaccinated fish challenged with *Aeromonas* spp. On the other hand, vaccinated fish showed fewer and less severe external and internal lesions following challenge with *Streptococcus* spp. and *Aeromonas* spp. compared with the unvaccinated group. The external lesions included hemorrhages in the skin involving the scales (Figure 4g) and bilateral exophthalmia (Figure 4h) following challenge with *Streptococcus* spp., while the brain appeared normal (Figure 4i) and the gall bladder was slightly swollen (Figure 4j). The vaccinated fish that were challenged with *Aeromonas* spp. exhibited hemorrhages in the skin and pectoral fin (Figure 4k), with normal livers and gall bladders (Figure 4l).

*Streptococcus* spp. were successfully isolated from the eye, brain, spleen, and liver of infected dead fish. Similarly, *Aeromonas* spp. were successfully isolated from the skin lesion, liver, kidney, and spleen. The presence of *S. agalactiae*, *S. iniae*, *A. hydrophila*, and *A. veronii* was confirmed by the 1500 bp band of 16S rRNA gene sequences (Figure 4m,n) and BLAST analyses.

#### 3.2.2. Protective Efficacies of the Feed-Based Bivalent Vaccine

The mortality and RPS percentages among unvaccinated (control) and vaccinated groups were observed 14 days after challenge. Results revealed that the unvaccinated fish began to die on day 1 and reached 100% mortality after day 10 post-challenge (Table 3). Vaccination with the feed-based bivalent vaccine significantly (*p* ≤ 0.05) enhanced fish resistance to infection, resulting in a high RPS value. The RPS values for the vaccinated group challenged with *S. agalactiae* and *A. hydrophila* were 80.00 ± 10.00% and 90.00 ± 10.00%, respectively. In addition, the RPS values for vaccinated fish challenged with *S. iniae* and *A. veronii* were 63.33 ± 5.77% and 60.00 ± 10.00%, respectively (Table 3).

#### 3.2.3. Histopathological Changes in Infected Fish

**Figure 5:**
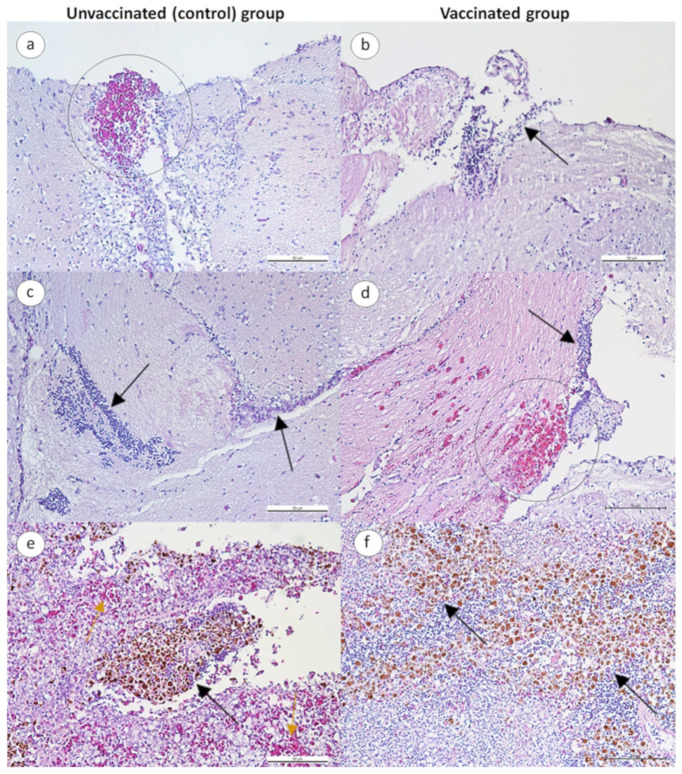
Photomicrograph of the brain and spleen of unvaccinated and vaccinated red hybrid tilapia following challenge with *Streptococcus* spp. (HE, bar = 50 μm). (**a**–**c**) The brain and spleen of unvaccinated fish, and (**d**–**f**) the brain and spleen of vaccinated fish. (**a**) Hemorrhage and mononuclear cell infiltration (black circle). (**b**) Infiltrations of mononuclear cells in the cerebrum. (**c**) Infiltrations of mononuclear cells in the cerebrum of unvaccinated fish. (**d**) Congestion and hemorrhages (black circle), and infiltrations of mononuclear cells in the meninges (black arrow). (**e**) Melano-macrophage aggregates (black arrow) and congestion in the spleen (yellow arrow). (**f**) Melano-macrophage aggregates in the spleen (black arrow).

In general, both vaccinated and unvaccinated fish challenged with *Streptococcus* spp. showed similar histopathological changes in the brain, wherein congestion and hemorrhages, and the infiltration of mononuclear cells, were observed in the cerebrum and meninges (Figure 5a,b,d,e). The spleen of unvaccinated fish showed the congestion of blood vessels, cytoplasmic vacuolations in some cells, and melano-macrophage aggregates (Figure 5c), which was less severe in vaccinated fish (Figure 5f).

**Figure 6:**
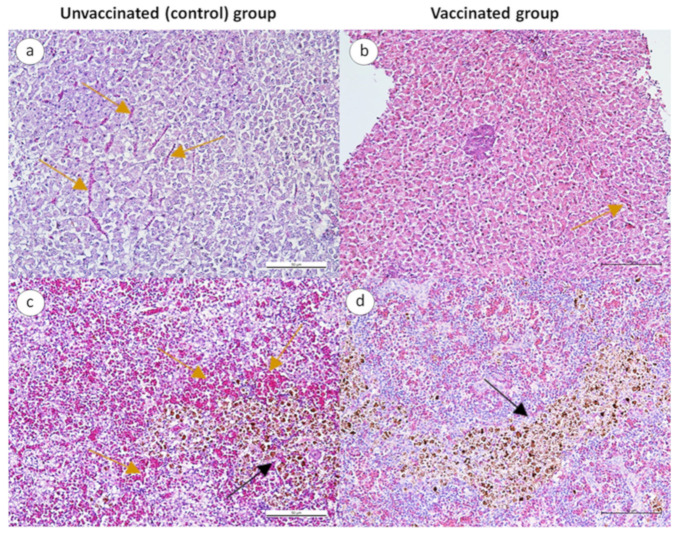
Photomicrograph of the liver and spleen of unvaccinated and vaccinated red hybrid tilapia following challenge with *Aeromonas* spp. (H & E stain, bar = 50 μm). (**a**) Vascular congestion (yellow arrow) in the liver of unvaccinated fish. (**b**) Mild congestion in the liver of vaccinated fish. (**c**) Melano-macrophage aggregates (black arrow) and congestion (yellow arrow) in the spleen of unvaccinated fish. (**d**) Melano-macrophage aggregates (black arrow) in the spleen of vaccinated fish.

Following the challenge with *Aeromonas* spp., the livers and spleens of unvaccinated fish showed congestion and mild hemorrhage (Figure 6a,c), whereas vaccinated fish showed milder congestion (Figure 6b,d).

### 3.3. Immunological Responses

#### 3.3.1. Lysozyme Activity

**Figure 7:**
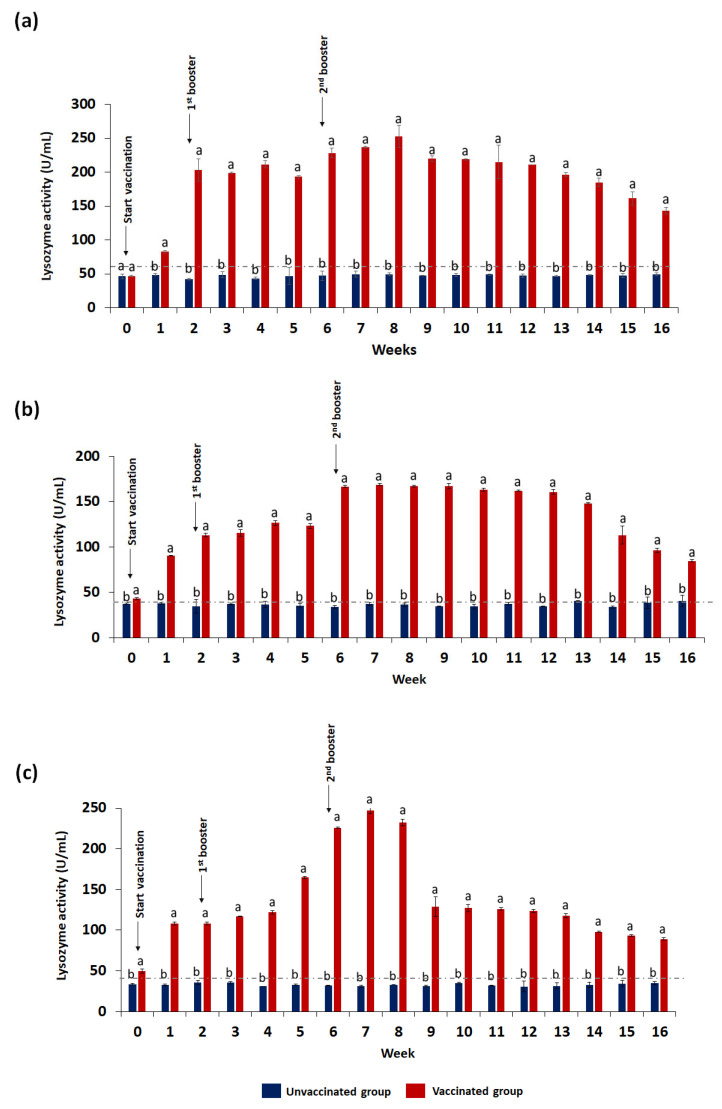
Lysozyme activity in serum (**a**), gut lavage fluid (**b**), and skin mucus (**c**) of red hybrid tilapia collected per time point (*n* = 3). The values are the mean ± standard deviation in triplicate samples. One-way ANOVA was performed with the Tukey–Kramer post hoc test to compare lysozyme activity between vaccinated and unvaccinated groups. Means with different superscript letters (a–b) differ significantly at *p* ≤ 0.05 (one-way ANOVA) significance level.

Lysozyme activities of unvaccinated (control) and vaccinated were determined in serum (Figure 7a), gut lavage (Figure 7b), and skin mucus (Figure 7c). The activity in all samples of the vaccinated fish was significantly (*p* ≤ 0.05) higher than the unvaccinated fish as early as week 1, with significant (*p* ≤ 0.05) incremental patterns, especially following vaccination. The highest activity in serum, gut lavage, and skin mucus of the vaccinated group was 252.75 ± 3.56 U/mg, 168.75 ± 2.84 U/mg, and 247.25 ± 2.54 U/mg, respectively.

#### 3.3.2. IgM Level

**Figure 8:**
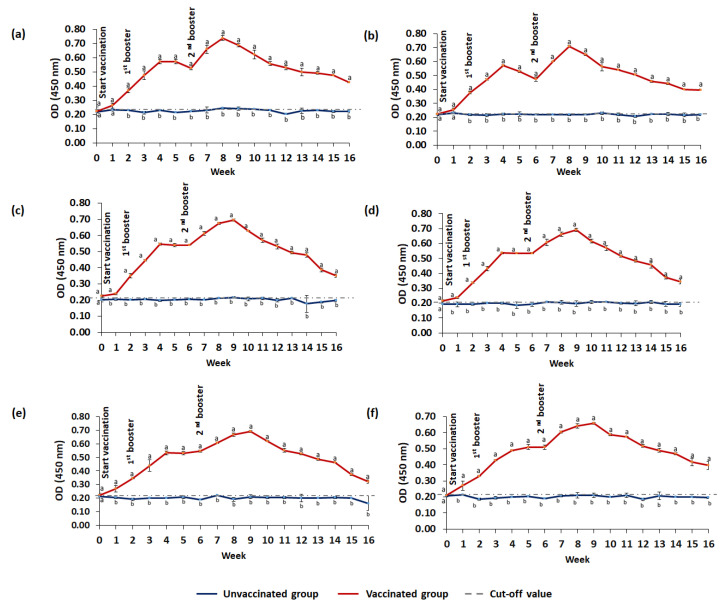
Specific IgM levels against *S. agalactiae* and *A. hydrophila* in red hybrid tilapia. The blue line represents the unvaccinated (control) group, and the red line represents the vaccinated group. Serum IgM levels against *S. agalactiae* (**a**) and against *A. hydrophila* (**b**). IgM levels against *S. agalactiae* in gut lavage (**c**) and against *A. hydrophila* (**d**). IgM levels in skin mucus against *S. agalactiae* in skin mucus (**e**) and against *A. hydrophila* (**f**). The number of fish used per time point (*n* = 6). Values are mean ± standard deviation in triplicate samples. One-way ANOVA was performed with the Tukey–Kramer post hoc test to compare IgM levels between vaccinated and unvaccinated groups. Means with different superscripts (a–b) differ significantly at *p* ≤ 0.05 (one-way ANOVA) significance level.

**Figure 9:**
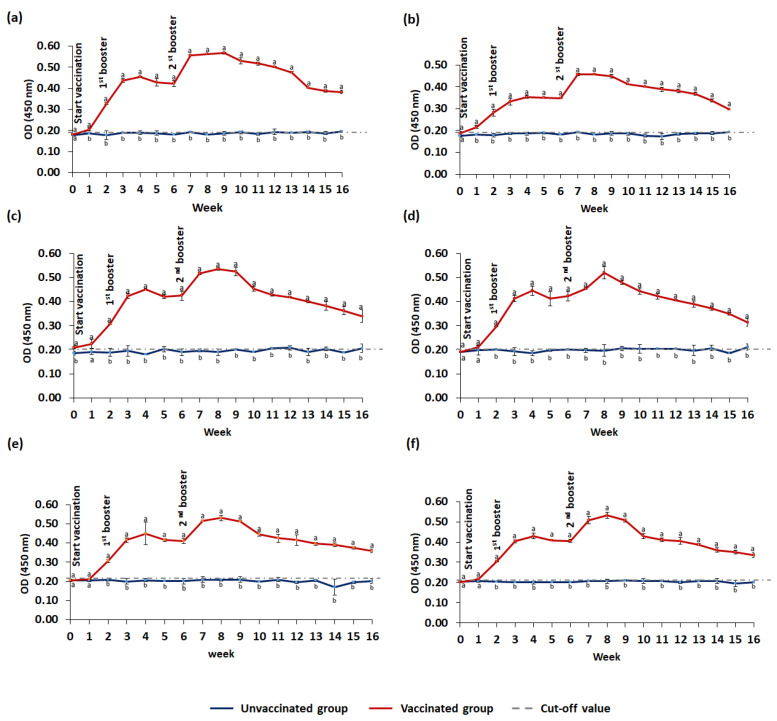
Specific IgM levels against *S. iniae* and *A. veronii* in red hybrid tilapia. The blue line represents the unvaccinated (control) group, and the red line represents the vaccinated group. Serum IgM levels against *S. iniae* (**a**) and against *A. veronii* (**b**). Gut lavage IgM levels against *S. iniae* (**c**) and against *A. veronii* (**d**). IgM levels in skin mucus against *S. iniae* (**e**) and against *A. veronii* (**f**). The number of fish used per time point (*n* = 6). Values are mean ± standard deviation in triplicate samples. One-way ANOVA was performed with the Tukey–Kramer post hoc test to compare IgM levels between vaccinated and unvaccinated groups. Means with different superscripts (a–b) differ significantly at *p* ≤ 0.05 (one-way ANOVA) significance level.

The IgM antibody titers against *S. agalactiae*, *A. hydrophila*, *S. iniae*, and *A. veronii* in the serum, gut lavage, and skin mucus of vaccinated and unvaccinated red hybrid tilapia are summarized in Figure 8 and Figure 9. Vaccinated fish produced significantly (*p* ≤ 0.05) higher antibody levels than unvaccinated fish with a cut-off level as early as week one, and remained significantly (*p* ≤ 0.05) high throughout the 16-week study period. Following administrations of booster doses, the IgM levels against *S. agalactiae* and *A. hydrophila* showed an increasing pattern and remained significantly high until week 9 (Figure 8). The highest IgM levels against *S. agalactiae* and *A. hydrophila* in the serum, gut lavage, and skin mucus were observed after the second booster. Similarly, the increasing IgM patterns against *S. iniae* and *A. veronii* were observed following booster doses (Figure 9), and the peak IgM levels against *S. iniae* and *A. veronii* were observed following the second booster.

## 4. Discussion

Streptococcosis and MAS are diseases that cause high mortality in aquaculture. Co-infections have also been reported, leading to high economic losses [6,11,13,28]. Antibiotics have been used to control disease outbreaks, but antibiotics might harm the environment and other living organisms [42,43]. Vaccination is considered a better alternative [24,44,45,46], and an oral delivery method offers more practicality [36]. Nevertheless, vaccines against streptococcosis and MAS using injection ad immersion delivery methods have been developed, although these target a single infection and are less practical [47]. Mortality in cultured fish is not caused by a single infection; therefore, developing a bivalent or cross-protective vaccine provides the advantage of protecting against multiple infections by different pathogens. This simultaneously provides practical ease of application and reduction in vaccination costs.

*Streptococcus agalactiae* has been identified as the most common bacterium that causes streptococcosis, although *S. iniea* has also been implicated in outbreaks of streptococcosis [25,41,48,49,50,51]. In addition to streptococcosis, MAS, which is caused mainly by *A. hydrophila*, is responsible for great economic losses in the aquaculture sector [52]. The present study used a feed-based bivalent vaccine containing formalin-killed *S. agalactiae* and *A. hydrophila* that was supplemented with 10% palm oil before being incorporated into fish pellets. These modifications did not significantly alter the nutrient compositions that are required for healthy and high-quality fish farming production [53], even though carbohydrates, moisture, proteins, and total ash were found to be significantly (*p* ≤ 0.05) reduced, while total fat and energy were significantly (*p* < 0.05) higher compared with the commercial tilapia pellet. Nevertheless, the nutrient compositions of the feed-based bivalent vaccine were found within the standard range of tilapia feed compositions as provided by the Food and Agriculture Organization [54]. Feed mixing and pelleting processes might affect the feed-based bivalent vaccine nutrient compositions [46]. The higher energy and total fat contents might be due to the addition of 10% palm oil. Palm oil has been reported to contain high levels of fat [55], which potentially gives adequate energy for fish growth [56].

The good characteristics of fish feed include high acceptability and stability. The feed intake is crucial to avoid growth inhibition. According to Dong et al. [31], low palatability might reduce the feed intake, leading to the failure of nutrient or antigen delivery into the gut. The feed-based bivalent vaccine used in this study had high palatability and showed no significant (*p* ≥ 0.05) change compared with the commercial tilapia pellet after 1 h of feeding. This suggests that the feed-based bivalent vaccine containing palm oil did not affect the palatability and feed intake of red hybrid tilapia. In addition, the palm oil might act as an adjuvant that enhances cell-mediated immunity and antibody production. In addition to palatability, the physical stability of feed pellets in water plays an essential indicator of good feed. Feed pellets with low water stability might disintegrate quickly and the nutrients will leach into water before being ingested by fish, deteriorating the water quality [32]. Overall, physical stability test results suggested that the feed-based bivalent vaccine was stable in water, similar to the commercial tilapia pellet.

There was no significant difference (*p* ≥ 0.05) in the growth performance of tilapia following vaccination. This is because the weights of the vaccinated and unvaccinated fish in this study were statistically insignificant (*p* ≥ 0.05), suggesting that the vaccine did not negatively influence or reduce the growth performance. Studies on the vaccination of Nile tilapia against *S. agalactiae* delivered through injection also showed no significant differences (*p* ≥ 0.05) in growth performance [57,58].

Generally, vaccination enhances the immune response, both innate and systemic immunities. The lysozyme activity in the serum, gut lavage, and skin mucus was significantly (*p* ≤ 0.05) higher following oral vaccination, especially following booster dose. Lysozyme is involved in innate immunity and is crucial in mediating protection against bacterial infection [59] as the first line of defense that resists initial infection [60]. Similarly, the bivalent vaccine stimulated higher IgM levels against *S. agalactiae*, *A. hydrophila*, *S. iniae*, and *A. veronii* in the serum, gut lavage, and skin mucus as early as week one, suggesting that the feed-based bivalent vaccine could potentially provide cross-protection against both *S. iniae* and *A. veronii*. It seems that the bivalent vaccine strongly promotes both mucosal and systemic immune responses in fish [61]. These were translated to the fact that vaccination with the bivalent vaccine resulted in a higher survival rate and less severe clinical signs and pathological lesions following challenge [41,62].

## 5. Conclusions

The feed-based bivalent vaccine containing formalin-killed *S. agalactiae* and *A. hydrophila* with palm oil exhibits significant immune-protective potential against streptococcosis and MAS in red hybrid tilapia. The bivalent vaccine provided high efficacy against mortality by *S. agalactiae* and *A. hydrophila*, but not full protection. In fact, it also provided partial cross-protection against infections by *S. iniae* and *A. veronii*. The vaccine improves fish’s innate and systemic immune responses, and could be a promising vaccine against the causal agents of streptococcosis and MAS in red hybrid tilapia. This study suggests that the duration of protection from the vaccine is up to 16 weeks. For a longer culture period, another booster dose should be explored to extend the high level of immune protection in the host.

## Figures and Tables

**Table 1:** Proximate compositions of a commercial tilapia pellet and the formulated pellets. The standard dietary requirements for tilapia fingerlings (10–30 g) were provided by the FAO (2017).

Test Description	Standard Dietary	Commercial Tilapia Pellet	Formulated Pellet	
			Control	Bivalent Vaccine
Carbohydrate (%)	>25	44.26 ± 0.15 ^a^	40.90 ± 0.47 ^b^	39.57 ± 0.13 ^c^
Energy (%)	na	354.00 ± 0.00 ^b^	397.50 ± 2.12 ^a^	395.00 ± 0.00 ^a^
Moisture (%)	<10	7.87 ± 0.02 ^a^	6.08 ± 0.01 ^c^	7.73 ± 0.02 ^b^
Protein (%)	28–35	34.36 ± 0.02 ^a^	31.87 ± 0.12 ^b^	31.18 ± 0.04 ^c^
Total ash (%)	<16	9.17 ± 0.02 ^b^	9.31 ± 0.01 ^a^	9.10 ± 0.01 ^c^
Total fat (%)	6–13	4.36 ± 0.02 ^c^	11.85 ± 0.37 ^b^	12.43 ± 0.05 ^a^

Note: ‘na’ indicates not available. Values are the mean ± standard deviation in duplicate samples. Different superscript letters (a–c) within the same rows indicates a significant difference at *p* ≤ 0.05.

**Table 2:** Effects of the dietary inclusion of different treatments on red hybrid tilapia’s final weight, weight gain, specific growth rate (SGR), and feed conversion ratio (FCR). Values are the mean ± standard deviation in triplicate samples. Insignificant differences (*p* ≥ 0.05) between control and vaccinated group.

Parameters	Treatment Group	
	Group 1 Unvaccinated (Control)	Group 2 Vaccinated (Bivalent Vaccine)
Initial body weight (g)	12.94 ± 0.46	13.10 ± 0.72
Final body weight (g)	33.67 ± 0.98	35.83 ± 1.20
Weight gain (g)	20.73 ± 0.97	22.73 ± 1.83
SGR (%/day)	1.59 ± 0.49	1.68 ± 0.35
FCR (g/g)	1.97 ± 0.07	1.82 ± 0.10

**Table 3:** Mortalities and relative percentage survival (RPS) of red hybrid tilapia 14 days post-challenged with *Streptococcus* spp. or *Aeromonas* spp. Values are the mean ± standard deviation in triplicate samples. Different superscript letters (a–c) within the column indicate a significant difference at *p* ≤ 0.05.

Challenge Bacteria	Treatment Group	Challenge Dose (CFU/mL)	Dead/ Total	Mortality (%)	RPS (%)
*S. agalactiae*	Vaccinated	1.8 × 10^3^	6/30	20.00	80.00 ± 10.00 ^b^
	Unvaccinated		30/30	100.00	-
*A. hydrophila*	Vaccinated	2.0 × 10^4^	3/30	10.00	90.00 ± 10.00 ^a^
	Unvaccinated		30/30	100.00	-
*S. iniae*	Vaccinated	1.5 × 10^3^	11/30	36.66	63.33 ± 5.77 ^c^
	Unvaccinated		30/30	100.00	-
*A. veronii*	Vaccinated	1.8 × 10^4^	12/30	40.00	60.00 ± 10.00 ^c^
	Unvaccinated		30/30	100.00	-

## Data Availability

The data presented in this study are available on request from the corresponding author.
